# Canonical and emerging regulatory mechanisms of tissue remodeling: shared principles across organs and therapeutic opportunities

**DOI:** 10.3389/fimmu.2026.1842892

**Published:** 2026-06-05

**Authors:** Meiyu Wu, Zichao Han, Wenhao Wei, Yewen Niu, Hao Wang, Fanchao Meng, Qingmei Wu

**Affiliations:** 1 Jinan Mingshui Eye Hospital, Jinan, Shandong, China; 2 The People’s Hospital of ZouPing City, Binzhou, Shandong, China

**Keywords:** fibrosis, mechanisms, signaling networks, review, tissue remodeling

## Abstract

Tissue remodeling refers to the structural and functional alterations an organ undergoes in response to changing environmental conditions. This process is precisely regulated by complex signaling networks. While these networks are essential for physiological repair, their dysregulation can lead to pathological conditions. Traditionally, this process is driven by a core set of molecular pathways, including the TGF-β superfamily, growth factors, and the MMPs/TIMPs system. These pathways primarily influence extracellular matrix turnover and fibroblast activation. The resulting inflammatory microenvironment is further modulated by changes in the Th1/Th2 balance and macrophage polarization. Recent studies, however, have highlighted the critical role of emerging regulatory networks. Mechanical signals are now understood to be transduced through the integrin-YAP/TAZ axis. Additionally, metabolic reprogramming, mediated by the glycolysis-HIF-mTOR axis, supplies the necessary energy for cell activation. Non-coding RNAs further refine these processes through multi-level regulation. This review synthesizes both canonical and emerging mechanisms. Our aim is to uncover the shared principles underlying pathological remodeling in different organs, providing a theoretical foundation for the development of precision intervention strategies across diseases.

## 1 Conceptual framework of tissue remodeling in physiology and pathology

Tissue remodeling refers to the structural, compositional, and functional alterations that a specific tissue or organ undergoes in response to internal or external environmental changes during growth, adaptation, or disease (1). Under physiological conditions, this process contributes to the maintenance of tissue homeostasis. In the presence of persistent or abnormal stimuli, however, tissue remodeling can evolve into a central mechanism driving the initiation and progression of chronic diseases. Such aberrant remodeling is often characterized by sustained alterations in tissue structure and mechanical properties, ultimately resulting in organ dysfunction. Consequently, tissue remodeling represents a major challenge in the management of chronic diseases and remains a key focus of current research (2). In chronic respiratory inflammatory diseases, for example, prolonged inflammation induces interactions among airway epithelial cells, smooth muscle cells, and immune cells. These interactions promote airway wall thickening and luminal narrowing, thereby impairing lung function (3). Elucidation of the molecular mechanisms underlying tissue remodeling, particularly in the context of chronic disease, is therefore essential for the development of effective therapeutic strategies. Although remodeling processes differ among organs and tissues, considerable similarities exist in their regulatory mechanisms. Evidence indicates that core pathways, including activation of TGF-β signaling and imbalance between MMPs and TIMPs, play pivotal roles in remodeling across multiple tissues (4, 5). Systematic and in-depth investigation of these shared mechanisms may provide new perspectives for interdisciplinary research while facilitating the identification of potential therapeutic targets and innovative intervention strategies. In this review, classical mechanisms refer to well-established and widely recognized regulatory pathways in tissue remodeling, including TGF-β family signaling, ECM metabolic imbalance mediated by MMPs/TIMPs, immune and inflammatory responses, and the pro-fibrotic effects of growth factors such as VEGF and PDGF. Emerging mechanisms, in contrast, are regulatory pathways that have been increasingly recognized in recent years but remain under active investigation, encompassing matrix stiffness–dependent mechanotransduction, cellular metabolic reprogramming, and epigenetic regulation, including ncRNAs. The present review aims to synthesize the common molecular mechanisms underlying tissue remodeling and fibrosis across different research fields. Future research directions will also be discussed to provide a broader perspective for both researchers and clinicians. Ultimately, this work seeks to advance understanding of pathological remodeling and support the development of novel therapeutic strategies.

## 2 Shared mechanisms of cross-organ tissue remodeling

Despite structural and functional differences among organs, pathological tissue remodeling exhibits common cross-organ mechanisms. Persistent inflammation often initiates remodeling, with immune cells such as monocytes and macrophages infiltrating injured sites over prolonged periods and releasing pro-fibrotic cytokines that drive cellular activation (6). Within this inflammatory microenvironment, fibroblasts play a central role: quiescent fibroblasts transform into highly secretory, proliferative myofibroblasts that produce large amounts of collagen and other ECM components (7). Under physiological conditions, ECM synthesis and degradation are tightly regulated by MMPs and TIMPs. Pathologically, this balance is disrupted. ECM synthesis remains elevated while degradation is suppressed, resulting in ECM accumulation (8). This not only occupies tissue space but also increases tissue stiffness and alters ECM physical properties. The stiffened matrix activates mechanosensitive pathways, including YAP/TAZ and Rho/ROCK, through integrins and FAK, further promoting fibroblast activation and ECM deposition. This creates a positive feedback loop of matrix-dependent mechanotransduction (9). Fibroblast activation is also accompanied by metabolic reprogramming. These metabolic changes both reflect the activation state and provide energy and substrates necessary for sustained ECM synthesis, directly linking cellular metabolism to tissue remodeling (10). Epigenetic regulation, especially by ncRNAs, further contributes to remodeling. ncRNAs regulate gene expression by interacting with multiple signaling pathways to control cell proliferation, differentiation, migration, and fibrotic responses, effectively bridging metabolic alterations and stable phenotypic changes (11). Collectively, persistent inflammation, ECM imbalance, mechanotransduction, metabolic reprogramming, and ncRNA-mediated epigenetic regulation constitute the shared mechanisms driving pathological tissue remodeling across different organs.

## 3 Classical mechanisms of tissue remodeling

### 3.1 TGF-β superfamily

#### 3.1.1 TGF-β subfamily smad signaling of TGF-β

TGF-β is a multifunctional cytokine that plays a central role in tissue remodeling after injury. It activates mesenchymal cells, such as fibroblasts, to produce and secrete matrix proteins like collagen and fibronectin, while also inhibiting matrix degradation by balancing MMPs and TIMPs (12, 13). This dual regulation promotes matrix synthesis and reduces its breakdown, leading to ECM deposition and remodeling. These changes provide the structural framework for tissue repair. Additionally, TGF-β drives fibroblast differentiation into myofibroblasts, contractile and secretory cells that are key players in the remodeling process (7). However, sustained activation of TGF-β signaling leads to fibrosis and tissue stiffening, which can impair the function of vital organs, including the heart, lungs, kidneys, and liver. Understanding how TGF-β signaling contributes to pathological remodeling is crucial for identifying fibrotic mechanisms and potential therapeutic targets.

The TGF-β signaling pathway plays a complex role in scleral remodeling in myopia. Contrary to its profibrotic role in most organs, the TGF-β/Smad pathway in myopia suppresses collagen synthesis and accelerates ECM degradation, ultimately leading to scleral thinning and axial elongation (14–16). Notably, this effect is highly tissue-specific. All three TGF-β isoforms (β1, β2, and β3) are downregulated in the sclera but upregulated in the retina and choroid (15). Furthermore, only aqueous humor TGF-β levels, rather than systemic circulating levels, correlate with axial length (17), suggesting that local microenvironmental signals, rather than systemic factors, predominantly drive scleral remodeling in myopia. Mechanistically, TGF-β suppresses Col-I synthesis through inhibition of the transcription factor Sp1 (18). Simultaneously, it activates the complement system and the NF-κB/NLRP3 inflammatory pathway, thereby enhancing MMP-2 activity and accelerating collagen degradation (19, 20). This example demonstrates that the same signaling pathway can produce opposite remodeling outcomes depending on the tissue context, underscoring the importance of tissue specificity when applying cross-organ remodeling principles. The differential roles of TGF-β in asthma and COPD further reveal how the shared principles of persistent inflammation and fibroblast activation manifest in distinct forms under different disease contexts. In asthma, TGF-β binds to the serine/threonine kinase receptor TβRII on the cell membrane, leading to phosphorylation of TβRI and activation of the Smad2/3 pathway (21). The phosphorylated Smads form a complex with Smad4 and translocate to the nucleus, promoting the transcription of Col-I/III/IV and fibronectin, which results in increased ECM deposition and basement membrane thickening (22). TGF-β also induces EMT in airway epithelial cells, characterized by reduced E-cadherin expression, upregulation of mesenchymal markers, and enhanced cell migration, all of which activate fibroblasts and smooth muscle cells, promoting airway wall remodeling and hyperresponsiveness (4, 23). In COPD, however, the role of TGF-β exhibits a dual nature. Under mild injury conditions, TGF-β promotes epithelial repair and suppresses excessive inflammation (24). Following long-term smoke exposure, this pathway exhibits a paradoxical pattern characterized by both excessive activation, such as elevated TGF-β1 levels and enhanced Smad2/3 phosphorylation (25, 26), and localized signaling defects, including reduced TβRII expression in bronchial glands (27). These findings suggest that diminished local signaling may contribute to disease progression. The dual pattern of overactivation and localized signaling deficits highlights the complexity of TGF-β regulation in COPD.

**Figure 1:**
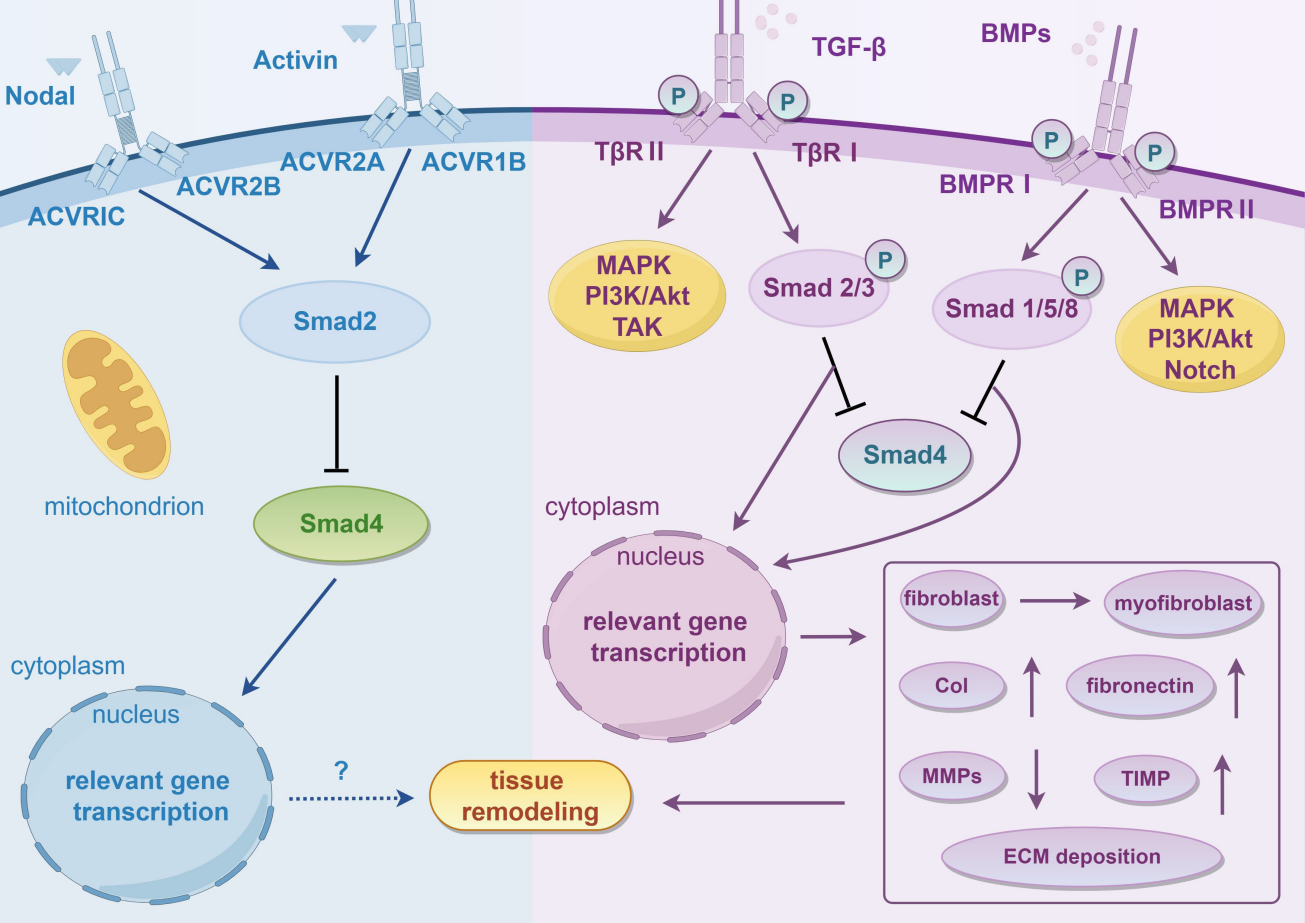
TGF-β and BMPs drive fibrosis and tissue remodeling by activating the Smad2/3 and Smad1/5/8 signaling pathways, respectively, binding with Smad4, and translocating into the nucleus to promote the transcription of related remodeling genes. Importantly, the ultimate effects of the BMP/Smad1/5/8 pathway in remodeling, whether pro-fibrotic or anti-fibrotic, are highly context-dependent and are determined by the specific BMP isoform, target tissue, and pathological condition. TGF-β and BMPs can also participate in the remodeling process by activating non-canonical pathways, such as MAPK, PI3K/AKT, and others. In contrast, the roles of the Activin/Nodal signaling pathway in tissue remodeling remain relatively underexplored, and its specific functions and regulatory mechanisms require further elucidation.

In summary, the TGF-β signaling pathway plays a pivotal role in physiological repair, pathological remodeling, and fibrosis. The role of the TGF-β superfamily in tissue remodeling is detailed in **Figure 1**. Therapeutic approaches targeting this pathway and its upstream or downstream signals have shown promising potential in various diseases. Future research should focus on understanding the dynamic interactions among these signaling components in complex microenvironments. Achieving precise, tissue-specific targeting of these pathways is essential for developing effective anti-fibrotic and anti-remodeling therapies.

#### 3.1.2 BMP/GDF subfamily

BMPs are multifunctional signaling molecules within the TGF-β superfamily. Initially recognized for their ability to induce ectopic bone and cartilage formation, further research has highlighted their crucial roles in embryonic development, tissue homeostasis, and remodeling (28, 29). More than 20 BMPs have been identified and classified into subfamilies based on their structural and functional characteristics. Among these, BMP-2, BMP-4, and BMP-9 have the strongest chondrogenic differentiation potential (30). GDF-5 promotes cartilage formation by enhancing cell adhesion and increasing skeletal element size, and also plays a key role in tendon healing (31, 32). BMP-3, the most abundant BMPs in bone tissue, promotes both bone formation and resorption by stimulating mesenchymal stem cell differentiation into osteoblasts, enhancing proliferation, and promoting bone matrix synthesis (33). In orthodontic tooth movement models, elevated BMP-3 expression correlates with increased osteoclast numbers and enhanced bone resorption (34). In contrast, BMP-13 inhibits osteogenic differentiation of mesenchymal stem cells and limits pathological bone formation (35). BMP-12 primarily targets soft tissues, promoting tendon-to-bone junction regeneration (36). Through precise regulation of cell differentiation and ECM metabolism, distinct BMPs isoforms collectively maintain dynamic remodeling in skeletal, cartilage, and tendon tissues.

As important members of the TGF-β superfamily, BMPs participate in remodeling processes across multiple organs, including the bone, eye, cardiovascular system, lung, and liver. However, their effects are highly dependent on the specific BMP isoform, target tissue, and disease context. Notably, even the same signaling pathway may produce completely opposite outcomes under different pathological conditions. In the skeletal system, BMPs bind to BMPRII on the target cell membrane, leading to activation of BMPRI and formation of a tetrameric receptor complex (37). The activated BMPRI subsequently phosphorylates R-Smad proteins, which form complexes that translocate into the nucleus to initiate transcription of osteogenic genes (38). In addition, BMPs regulate the balance between osteogenesis and osteoclastogenesis through the MAPK and PI3K/Akt signaling pathways (39) and are negatively regulated by factors such as Noggin, Smad6/7, and miRNAs (40). Under physiological conditions, this system maintains bone homeostasis; however, under pathological conditions, it may become aberrantly activated. For instance, tumor-derived BMP-4/7 can promote bone destruction (41), whereas BMP-2 can enhance bone repair (42). Notably, feedback regulation exists within the BMP signaling network. For example, a p38α-mediated feedback loop modulates BMP-2 expression to maintain the balance between bone resorption and formation (43). TAK1 influences both osteoclast differentiation and osteogenesis/chondrogenesis through Smad-dependent signaling pathways (44). In addition, TSG-6 can simultaneously bind BMP-2 and RANKL, thereby inhibiting bone erosion while promoting osteogenic differentiation under inflammatory conditions, highlighting the intrinsic bidirectional regulatory nature of bone remodeling (45). Dysregulation of this network is associated with various bone disorders, and elucidating its underlying regulatory logic provides a theoretical basis for therapies targeting bone metabolism. In ocular tissues, BMP expression displays a tissue-specific pattern. BMP-5 and BMP-7 are highly expressed in the cornea, whereas BMP-2 and BMP-4 are expressed at relatively low levels. Cells in the trabecular meshwork and optic nerve head both secrete BMPs and express their corresponding receptors. In the retina, BMP-2, BMP-4, and BMP-7 are the predominant isoforms (46). A similar distribution of BMPs and their receptors has also been observed in the sclera of guinea pigs and humans (47). BMPs also contribute to scleral morphogenesis by coordinating with other signaling pathways and are regulated by local mechanical forces (48). Taking scleral remodeling in myopia as an example, BMP-2 is significantly downregulated in form-deprivation myopia models, whereas BMP-2 normally promotes ECM synthesis (49). This reduction leads to decreased ECM production, scleral thinning, and axial elongation, supporting a mechanism based on imbalance between ECM synthesis and degradation. These findings provide a theoretical basis for targeting BMPs pathways as a therapeutic strategy for myopia intervention.

Extending the perspective to other organs, the functional divergence of BMP signaling becomes even more pronounced, and the role of BMPs cannot be simply reduced to pro-fibrotic effects. In the cardiovascular system, BMP-7 inhibits cardiomyocyte apoptosis and attenuates adverse remodeling (50, 51), whereas BMP-2 induces osteogenic differentiation of vascular smooth muscle cells and promotes vascular calcification (52, 53). In the lung, BMP-4 inhibits the progression of fibrosis, whereas BMP-2 promotes airway remodeling under smoke exposure (54, 55). While some BMP family members are upregulated in diseased tissues, their functional significance is still debated. For example, BMP-1 is upregulated in pulmonary fibrotic tissues, but gene knockout studies show it is not essential for pulmonary fibrosis in mice, suggesting its role may be species- or organ-specific (56). In liver and intestinal tissues, BMPs are critical for maintaining tissue homeostasis and regulating injury repair. Disruption of BMP-9 signaling may promote liver fibrosis (57). BMP signaling also plays a role in intestinal epithelial regeneration and repair through epithelial-mesenchymal microenvironment interactions (58). Even in ectopic fibrosis, such as capsular formation around breast implants, periostin drives fibroblast activation by upregulating BMP-1 (59). In summary, BMPs play extensive and complex roles in tissue remodeling across multiple organs. Their functions are influenced by differences among BMP isoforms, as well as the tissue microenvironment and disease context. This context-dependent regulation suggests that future BMP-based therapies should aim to preserve their protective functions while avoiding pathological remodeling effects.

#### 3.1.3 Activin/nodal subfamily

The Activin/Nodal subfamily represents an important branch of the TGF-β superfamily and primarily functions in embryonic development and cell fate determination (60). In terms of signal transduction, both Activin and Nodal utilize the same receptors to activate Smad2, which subsequently forms a complex with Smad4 and translocates to the nucleus to regulate gene expression (61). Due to their highly similar signaling mechanisms, they are commonly described collectively as the Activin/Nodal pathway. This pathway is essential for early embryonic axis formation, mesendoderm induction, and left–right asymmetry establishment (62). In human embryonic stem cells, Activin/Nodal signaling is tightly regulated. BRD9 modulates this pathway by influencing H3K27ac modification, and its deletion inhibits mesendoderm differentiation in human embryonic stem cells, supplementation with Activin A or NODAL can rescue this defect (63). During directed stem cell differentiation, modulation of Activin/Nodal signaling serves as a key strategy for lineage specification. For example, inhibition of the pathway combined with BMP-4 suppresses mesendoderm formation and promotes differentiation of human embryonic stem cells toward trophoblast lineages (64). Similarly, pathway inhibition in mouse induced pluripotent stem cells enhances BMP-4 signaling and promotes differentiation toward specific lineages (65). Dysregulation of the Activin/Nodal pathway is also closely associated with pathological conditions, particularly cancer. TBX3 overexpression can drive an autocrine TBX3-Activin/Nodal positive feedback loop, thereby maintaining cancer stem cell self-renewal (66). Despite extensive investigation in stem cell differentiation and cancer biology, the role of Activin/Nodal signaling in tissue remodeling remains poorly defined.

Current studies have mainly focused on the functions of Activin/Nodal signaling in embryonic development, cell fate determination, and stem cell regulation. Research addressing its roles in tissue repair, regeneration, and structural remodeling remains limited. Existing evidence largely concerns epigenetic regulation, including chromatin remodeling and histone modification-mediated control of gene expression (64). However, epigenetic alterations do not necessarily translate directly into structural tissue remodeling, and systematic investigation in this area is still lacking. Therefore, how Activin/Nodal signaling regulates tissue remodeling under physiological or pathological conditions remains an important question for future research.

### 3.2 MMPs and TIMPs

MMPs are zinc and calcium dependent endopeptidases that regulate tissue development and remodeling by degrading the ECM. They play central roles in both physiological repair and pathological changes (67). The MMP family includes collagenases, gelatinases, stromelysins, and membrane-type MMPs. Collagenases such as MMP-1, MMP-8, and MMP-13 degrade fibrillar collagens, while gelatinases like MMP-2 and MMP-9 target denatured collagens and basement membrane components. Stromelysins, including MMP-3, MMP-7, and MMP-10, act on a broad range of ECM substrates. Membrane-type MMPs, such as MMP-14, are anchored to the cell surface and regulate pericellular proteolysis. Each MMP class plays a distinct role in processes such as tissue remodeling, tumor invasion, angiogenesis, and cell migration (68). TIMPs are glycoproteins that serve as the primary physiological inhibitors of MMP activity. By reversibly binding to MMPs, they maintain a dynamic balance between ECM synthesis and degradation, precisely controlling MMP proteolytic activity (69).

The balance between MMPs and TIMPs is central to ECM homeostasis. However, the imbalance of MMPs/TIMPs manifests differently across diseases, ultimately determining the outcome of tissue remodeling. In asthma-associated airway remodeling, the imbalance between MMP-2/9 and TIMP-1 exhibits a time-dependent pattern. MMP-9 is elevated in the early phase of remodeling and contributes to airway structural damage. In the later stage, sustained upregulation of TIMP-1 inhibits collagenase activity, leading to excessive collagen deposition and progression of fibrosis (70). These findings indicate that the MMPs/TIMP imbalance evolves as the disease progresses. Moreover, MMP expression closely correlates with airway inflammation, creating a vicious cycle between inflammation and remodeling. Inflammatory mediators such as IL-5 and LPS disrupt the MMPs/TIMP balance by activating relevant signaling pathways, further promoting remodeling (71, 72). Thus, MMPs not only act as effectors in airway remodeling but also represent potential therapeutic targets for intervening in asthma-associated airway remodeling through their activity imbalance and interaction with inflammation. In patients with high myopia, elevated MMP-2 levels lead to excessive ECM degradation (73). In addition, MMP-2 activation regulated through pathways such as AREG-ERK1/2 and Wnt7b/β-catenin further degrades collagen, resulting in tissue thinning rather than fibrosis, thereby producing an outcome opposite to that observed in asthma (74, 75). In addition to scleral remodeling, MMP-2 is also involved in ciliary muscle remodeling in myopia, leading to disruption of ciliary muscle structural integrity (76). These findings suggest that MMP-mediated matrix metabolic imbalance may be a common mechanism driving coordinated structural changes across multiple ocular tissues during myopia development. In the cardiovascular system, MMP-7 is positively correlated with the severity of vascular remodeling, whereas MMP-19 and TIMP-3 exert protective effects (77). MMP-2 links hemodynamic stimuli to vascular structural changes by mediating the recruitment of reparative cells to sites of injury via the LIMK1/Cofilin pathway (78). In addition, studies have shown that even within the same molecule, MMP functions may be paradoxical. For instance, MMP-9 drives aberrant dendritic spine remodeling in traumatic brain injury (79), whereas its sustained elevation after cardiac surgery suggests that remodeling can persist independently of acute inflammation over the long term (80). Genetic studies have linked MMP-1 and MMP-8 gene polymorphisms to the risk of tibialis posterior tendinopathy (81). In the tumor microenvironment, dense fibrotic remodeling driven by MMP-3 creates physical barriers that impede chemotherapeutic drug delivery (82). Together, these studies demonstrate that MMPs and TIMPs function not only as executors of local matrix degradation and reconstruction but also as critical regulatory nodes within the pathological remodeling networks of multiple organs.

The MMPs/TIMP system functions as a core regulatory network maintaining ECM homeostasis. Imbalance within this system represents a common pathological hub underlying aberrant remodeling across diverse tissues. From the airways and sclera to cardiovascular structures, pulmonary interstitium, nervous tissue, and tendons, the balance between MMPs and TIMPs governs not only normal development and repair but also drives structural remodeling in diseases characterized by inflammation, fibrosis, degeneration, and malignancy. Depending on the pathological context, such remodeling may manifest as ECM degradation, deposition, or stiffening. Collectively, current evidence suggests that despite substantial differences in affected organs and clinical manifestations, local modulation of the MMPs/TIMP system may represent a shared therapeutic strategy for a range of chronic progressive structural remodeling diseases.

### 3.3 Immune and inflammatory regulation

**Figure 2:**
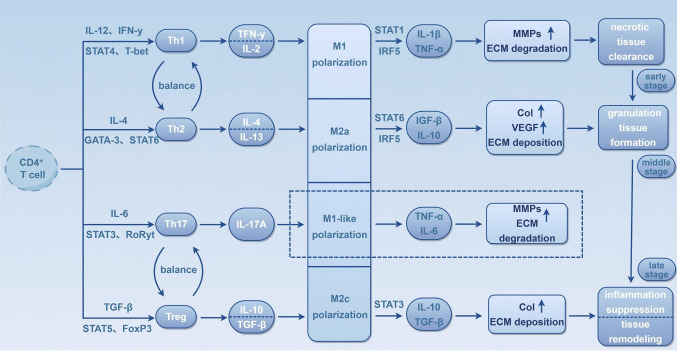
Th1 cells activate M1 macrophages by secreting factors such as IFN-γ, thereby promoting inflammatory responses and ECM degradation. Th2 cells secrete pro-inflammatory factors such as IL-4. however, the M2 macrophages regulated by these factors exert protective effects in tissue repair and immune tolerance through the secretion of anti-inflammatory factors. IL-17A enhances inflammatory responses by activating M1-like macrophage polarization. Treg cells regulate M2c macrophage polarization by secreting factors such as TGF-β and IL-10, and the synergistic interaction between these cells suppresses inflammatory responses and promotes tissue repair.

Tissue remodeling is a complex process involving dynamic ECM alterations, cellular phenotype transitions, and structural reorganization, all regulated by diverse signaling inputs. Among these factors, inflammation acts as both a critical trigger and a driving force, exerting dual roles in the initiation and progression of remodeling (83). Appropriate inflammatory responses initiate and guide physiological repair, whereas persistent inflammatory stimuli or dysregulated inflammatory networks promote pathological remodeling. Such pathological remodeling is characterized by excessive ECM deposition and distorted tissue architecture, ultimately leading to fibrosis and organ dysfunction (84). The detailed mechanism is shown in **Figure 2**. In discussing immune regulation, the classical Th1/Th2 and M1/M2 polarization models provide a useful conceptual framework for understanding the directional nature of immune responses in tissue remodeling. However, these dichotomous models represent a simplification of the highly complex phenotypes and functions of immune cells. Macrophages, in particular, can exhibit multiple mixed or distinct activation states under different microenvironmental conditions. Therefore, the Th1/Th2 and M1/M2 frameworks discussed below should be regarded as guiding working models rather than comprehensive representations of immune heterogeneity *in vivo*.

#### 3.3.1 Th1/Th2 and Th17/Treg balance

Th1 and Th2 cells are two major subsets of CD4^+^ T lymphocytes that mediate cellular and humoral immunity, respectively. These subsets counterbalance each other by secreting distinct cytokine profiles, playing crucial roles in immune responses. Th1 cells primarily secrete IFN-γ, which activates macrophages for cell-mediated immunity, while Th2 cells produce cytokines like IL-4 and IL-5 that promote B cell proliferation and antibody production (85). The balance between these subsets is essential for proper immune function, and its disruption drives various immune-related tissue remodeling processes. In asthma, Th2 responses predominate, with IL-4 and IL-13 promoting airway inflammation, mucus secretion, and fibroblast activation through the STAT6/GATA3 pathway, ultimately leading to airway wall thickening and fibrosis. In contrast, IL-5 mainly affects eosinophil differentiation, but alone is insufficient to drive pathological remodeling changes (86–88). In contrast, emphysema is characterized by Th1 predominance, in which IFN-γ induces alveolar epithelial cell apoptosis through a cathepsin S-dependent pathway, disrupts the protease/antiprotease balance, and drives alveolar destruction and remodeling (89, 90). Atopic dermatitis represents another phenotype of Th2 polarization, in which IL-4 and IL-13 promote sebaceous gland lipogenesis and proliferation through STAT6 signaling, leading to glandular remodeling (91). Collectively, these studies demonstrate that the direction of Th1/Th2 imbalance determines the type of remodeling. Th2 predominance leads to fibroproliferative remodeling, while Th1 predominance drives tissue-destructive remodeling. Restoring Th1/Th2 balance may therefore be a potential strategy for intervening in immune-related tissue remodeling processes. Although the Th1/Th2 model effectively explains the directional nature of remodeling in certain diseases, T-cell functions in many inflammatory microenvironments are far more complex than this binary paradigm. Interactions among other subsets, such as Th17 and Treg cells, are also critically important.

Similarly, the balance between Th17 and Tregs forms another critical immunoregulatory axis within the CD4^+^ T cell lineage. Th17 cells initiate inflammation by secreting proinflammatory cytokines such as IL-17, while Tregs limit excessive inflammation and maintain immune tolerance through inhibitory factors like IL-10 and TGF-β (92). Like Th1/Th2 imbalance, Th17/Treg dysregulation plays a pivotal role in various immune-related pathological processes, particularly in the transition from inflammation to structural remodeling. In asthma, enhanced Th17 differentiation and impaired Treg function lead to increased IL-17A release, thereby exacerbating airway inflammation, hyperresponsiveness, and remodeling (93). In lipopolysaccharide-aggravated models, IL-17 further promotes vascular remodeling through synergistic activation of Th2/Th17 pathways (94). Similarly, in the vascular system, Th17/Treg imbalance drives inflammatory vascular remodeling, whereas reduced Treg activity impairs anti-inflammatory and reparative responses (95). These findings suggest that Th17/Treg imbalance primarily sustains pathological remodeling by amplifying inflammation and disrupting normal tissue repair processes. Therefore, restoring this balance may hold therapeutic potential across multiple organs, although its efficacy should be evaluated in conjunction with other factors within the local microenvironment.

#### 3.3.2 M1 and M2 macrophage polarization

M1 macrophages play a critical role in tissue remodeling, with their polarization state determining the direction of immune responses and tissue repair. In response to microenvironmental signals, macrophages can polarize into distinct phenotypes, primarily proinflammatory M1 and anti-inflammatory M2 subtypes (96). M1 macrophages drive inflammatory responses and participate in pathogen clearance through the secretion of proinflammatory cytokines such as TNF-α, IL-1β, and IL-6. However, excessive M1 activation can lead to tissue damage and fibrosis. For example, CD11b exacerbates hypertensive cardiac remodeling by promoting M1 macrophage polarization (97). TNF-α promotes tissue degeneration and inflammation via the MAPK pathway, contributing to capillary-to-vein vascular remodeling in Mycoplasma pneumoniae infection, matrix degradation in cardiomyopathy, and vascular wall thickening in pulmonary arterial hypertension (98–101). IL-1β, a classic proinflammatory cytokine, drives fibrotic remodeling by influencing fibroblast immune transdifferentiation (102). Studies show that IL-1β neutralizing antibodies reduce collagen deposition and downregulate fibrosis-associated molecules, including TGF-β, TIMP-1, and MMP-2, in myocarditis models, confirming its role in the transition from inflammatory myocardial injury to adverse remodeling (103). IL-6 participates in remodeling primarily through trans-signaling pathways. In periodontitis, activation of the IL-6/JAK2/STAT3 pathway increases the RANKL/OPG ratio, thereby exacerbating osteoclast-mediated bone resorption (104). In myopia, IL-6 secreted by the extraocular muscles upregulates scleral MMP-2 through trans-signaling, leading to ECM degradation and axial elongation (105). These findings suggest that targeting M1 macrophage polarization or their specific proinflammatory cytokines may provide a strategy for intervening in pathological remodeling. It should be noted that M1 polarization represents only one classical manifestation of the pro-inflammatory functions of macrophages. In reality, the M1/M2 dichotomy is a simplified representation of the complex activation states of macrophages, which often exhibit mixed phenotypes *in vivo*. This underscores the considerable plasticity and functional diversity of macrophages beyond a binary classification system.

Unlike M1 macrophage polarization, M2 macrophages are primarily characterized by promoting anti-inflammatory responses and tissue repair, playing significant roles in ECM remodeling and regenerative repair. However, in-depth analysis reveals that M2 polarization and its effector molecules exhibit functional duality across different pathological contexts, with their reparative effects potentially transitioning into pathological remodeling. In myocardial infarction repair, quercetin promotes M2 macrophage polarization through metabolic reprogramming, thereby alleviating fibrosis and improving cardiac remodeling (106). These findings suggest that the reparative functions of M2 macrophages can be therapeutically harnessed. In contrast, in hypoxic pulmonary arterial hypertension, chronic hypoxia induces IL-21-mediated M2 polarization of alveolar macrophages, which promotes pulmonary artery smooth muscle cell proliferation and pyroptosis, thereby aggravating pulmonary vascular remodeling (107). Thus, the same M2 polarization state can produce completely opposite remodeling outcomes under different microenvironmental conditions. The M2-derived effector molecule IL-10 also exhibits bidirectional effects. On the one hand, IL-10 attenuates vascular remodeling in hypertensive rats by suppressing TGF-β expression and perivascular inflammation (108). It also improves ventricular remodeling by promoting M2 polarization and downregulating Hyal3 expression, thereby optimizing ECM homeostasis (109). On the other hand, in systemic lupus erythematosus–associated pleural remodeling, IL-10 induces collagen deposition and pleural thickening in pleural mesothelial cells through activation of the JAK2/STAT3/HIF1α signaling axis (110). Moreover, transplantation of tumor-derived MDSCs reduces IL-10 levels and ameliorates airway remodeling, further suggesting that IL-10 may contribute to pathological remodeling under specific conditions (111). In summary, M2 macrophages and their effector molecule IL-10 play dual roles in tissue remodeling. They can inhibit pathological remodeling through anti-inflammatory repair mechanisms while also driving fibrosis and structural destruction under specific conditions. This duality underscores the need for precise intervention strategies that differentiate the directional effects of M2 macrophage polarization in various disease contexts. It should be emphasized again that the M1/M2 dichotomy represents a simplified model, as macrophages *in vivo* frequently exhibit mixed phenotypes. Accordingly, M2 should be regarded as a functional spectrum or directional state rather than a fixed subtype.

#### 3.3.3 Additional inflammatory pathways

The MAPK signaling pathways (ERK1/2, p38, JNK) constitute a conserved cascade that transduces extracellular signals, such as inflammation and mechanical stress, into nuclear transcriptional responses (112, 113). In tissue remodeling, their central function is to link upstream inflammatory cues to downstream fibroblast activation and ECM metabolism. For example, in cardiac remodeling, p38 activation promotes the transdifferentiation of fibroblasts into myofibroblasts (114). In intestinal fibrosis, the MAPK/ERK pathway mediates TGF-β1–induced ECM deposition, and its inhibition can restore the MMPs/TIMPs balance (115). In asthma models, p38 MAPK inhibition alleviates collagen deposition and mucus secretion (116). Collectively, evidence from these diverse organs indicates that MAPK serves as a critical bridge between inflammation and tissue remodeling. The cooperation between MAPK and NF-κB further amplifies these effects. In heart failure, their synergistic activation promotes cardiomyocyte hypertrophy, fibrosis, and inflammation, while inhibition of this axis reduces pathological remodeling (117). In asthma models, increased phosphorylation levels of p38 and ERK collectively drive inflammatory infiltration and airway injury (118). This coordinated signaling network enables cells to integrate multiple stress signals and determine both the intensity and duration of tissue remodeling. The NLRP3 inflammasome, as a sensor of innate immunity, also participates in tissue remodeling. After myocardial infarction, cardiac fibroblasts release vesicles containing damaged mitochondrial components, which activate NLRP3 and exacerbate inflammation and ventricular remodeling (119). In osteoarthritis, upregulation of ACSL1 enhances autophagolysosomal activity, suppresses NLRP3 activation, restores the balance between osteogenesis and osteoclastogenesis, and improves bone remodeling (120). Collectively, these findings indicate that NLRP3 activation contributes to the shift of tissue remodeling toward a pathological state. In summary, analyzing these signaling pathways at different stages of remodeling helps elucidate the regulatory switches governing the transition from physiological repair to pathological remodeling, providing a foundation for developing targeted interventions against organ fibrosis.

### 3.4 Growth factor families

TGF-β is a central driver of tissue remodeling, but its effects are modulated through synergistic interactions with other growth factors. These factors fine-tune TGF-β-mediated cellular responses, collectively influencing the final remodeling outcome. This section explores the roles of these growth factors in tissue remodeling.

#### 3.4.1 VEGF

The VEGF family (VEGF-A/B/C/D and PlGF) participates in pathological structural remodeling across multiple organs by activating VEGFR1–3 and downstream PI3K-Akt and MAPK signaling pathways (121). Their effects are highly context-dependent, as the same molecule can drive adaptive angiogenesis or lymphangiogenesis in different diseases or at different stages of the same disease, or alternatively exacerbate barrier disruption and fibrosis. In a pancreatitis model, VEGF-A knockout suppresses vascular proliferation but does not affect fibrosis, suggesting that its role is largely restricted to adaptive vascular remodeling (122). In viral myocarditis, VEGF-C promotes lymphangiogenesis via VEGFR3 and improves cardiac function (123). In contrast, in metabolic syndrome, impaired VEGF signaling leads to defects in myocardial angiogenesis and aggravates remodeling (124). In tumors, RIP1 upregulates VEGF-C through NF-κB signaling, driving lymphatic remodeling and promoting tumor progression (125). Overall, these studies highlight that therapeutic strategies targeting VEGF pathways must account for their context-dependent roles in various disease settings. Context-aware targeting provides a foundation for developing precision therapies.

In chronic inflammatory airway diseases, aberrantly elevated VEGF expression directly links inflammation to structural remodeling. In asthma, VEGF-A accelerates airway remodeling via the MAPK pathway, disrupts epithelial barrier integrity, and induces EMT (126, 127). Serum VEGF levels are positively correlated with disease severity and negatively correlated with lung function, suggesting its potential as a biomarker of inflammation–remodeling transition (128). In COPD, VEGF promotes pathological angiogenesis through the PI3K/Akt/mTOR pathway by upregulating VEGFR2 and ET-1 (129). Notably, in asthma patients with bronchiectasis, both VEGF and TGF-β1 are elevated in sputum, indicating a synergistic role in exacerbating tissue remodeling (130). In ocular diseases, VEGF signaling is even more complex. In normal sclera, VEGF signaling is actively suppressed to maintain an avascular state and immune privilege (131). Untreated ROP eyes exhibit a paradoxical combination of increased corneal endothelial cell density and reduced corneal thickness. However, these parameters tend to normalize in more severely affected eyes, suggesting that VEGF plays a role in complex, dynamic long-term adaptive changes in the corneal endothelium (132). In the treatment of neovascular age-related macular degeneration, differential effects of anti-VEGF therapies on choroidal remodeling, as well as the potential for anti-angiogenic treatment to induce progressive subretinal fibrosis, highlight a potential trade-off between angiogenesis inhibition and fibrotic remodeling (133, 134). In addition, metabolomic profiling of aqueous humor in patients with macular edema reveals that VEGF activity is associated with broad metabolic dysregulation, suggesting that VEGF functions not only as an angiogenic regulator but also as a modulator of the metabolic microenvironment (135). The VEGF family, through its receptors and downstream signaling pathways, can drive either adaptive angiogenesis or lymphatic remodeling in various pathological contexts. Alternatively, it may contribute to barrier disruption, fibrosis, and changes in the metabolic microenvironment. These findings highlight the complexity of VEGF signaling and lay a crucial foundation for developing precision-targeted therapeutic strategies.

#### 3.4.2 FGF

The FGF family plays complex and critical roles in both tissue homeostasis and pathological remodeling. Comprising 23 members, it is divided into paracrine and endocrine subfamilies based on secretion modes and functions. These growth factors are essential for physiological processes such as development, repair, and metabolic regulation, with dysregulation linked to various diseases (136, 137). FGF signaling is highly dependent on the tissue microenvironment, disease context, and interactions with other pathways, showcasing significant context dependence and functional diversity. In some conditions, FGF signaling has protective effects. For instance, in the sclera, exogenous FGF-2 promotes fibroblast proliferation and reverses fibrotic phenotypes, suggesting its role in maintaining scleral cell homeostasis and preventing abnormal remodeling (138). FGF signaling also supports myocardial growth and angiogenesis during embryonic heart development, and contributes to vascular remodeling in chronic kidney disease through the FGF-23/Klotho axis (139, 140). In the tumor microenvironment, FGF-2 promotes abnormal maturation of tumor vasculature, aiding tumor growth (141). During lactation, sharply elevated circulating FGF-21 specifically promotes bone resorption, leading to physiological bone loss and positioning FGF-21 as a key regulator of bone remodeling (142). The effectiveness of FGF signaling often depends on synergistic interactions with other signals, such as VEGF. For example, FGF-2 alone is insufficient to effectively promote angiogenesis and bone formation, suggesting that its effects require signals like VEGF (143). This contrasts with the role of FGF-9 in diabetic bone defects, highlighting how different pathological microenvironments influence FGF signaling outcomes (144). In summary, the FGF family regulates diverse physiological and pathological processes through complex signaling networks. Its dual roles result from extensive interactions with the microenvironment and other key pathways. Future research should focus on understanding how these factors and molecular switches determine the final effects of FGF signaling, providing a foundation for precision therapeutic approaches.

#### 3.4.3 PDGF

The PDGF family signals through five dimeric ligands formed by four subunits, which bind to their corresponding receptors and activate downstream signaling networks (145, 146). The effects of PDGF are highly dependent on ligand isoforms, cellular sources, target tissues, and interactions with other signaling pathways. In asthma-associated airway remodeling, PDGF-BB drives pathological changes through multiple mechanisms. It upregulates STOML2 and promotes inflammation and epithelial–mesenchymal transition via the p38 MAPK pathway (147). It also regulates TEP1 stability through METTL3-mediated m6A methylation (148). In contrast, TRIM33 acts as an endogenous negative regulator; its overexpression reverses the pro-remodeling effects of PDGF-BB by inhibiting Smad4 and Wnt/β-catenin signaling (149). Mast cells, eosinophils, and airway epithelial cells all secrete PDGF, collectively promoting smooth muscle proliferation and collagen synthesis (150). These regulatory layers highlight the therapeutic potential of targeting PDGF signaling. Beyond airway remodeling, PDGF plays a crucial role in pathological remodeling across multiple systems, including the cardiovascular, urinary, and vascular tissues. Its effects are highly dependent on tissue type, cellular source, and interactions within signaling networks. In cardiac remodeling, PDGF promotes fibroblast activation through the Smad2/3, JNK, and β-catenin pathways and synergizes with TGF-β to drive fibrosis (151). In heart failure, cardiac mesenchymal stem cells differentiate into profibrotic myofibroblasts via autocrine and paracrine PDGF signaling, creating a vicious cycle that can be interrupted by targeting PDGF (152). In obesity-associated hypertensive cardiac remodeling, macrophage-derived uPA cleaves the precursor of adipocyte-secreted PDGF-D, linking metabolism, inflammation, and cardiac fibrosis (153). In vascular remodeling, PDGF promotes smooth muscle cell migration and neointima formation through the IQGAP1-ATP7A-Rac1 axis (154). In summary, PDGF serves as a central hub in multi-organ pathological remodeling. Future research should focus on elucidating its dynamic signaling networks across different disease stages and cell types, while exploring optimized combinations with anti-inflammatory and anti-fibrotic strategies to achieve precision interventions.

#### 3.4.4 Others

CTGF exerts its effects in fibrosis and tissue remodeling through interactions with ECM components, receptors, and growth factors via its modular structural domains (155). However, its function is not exclusively pro-fibrotic but is highly dependent on disease stage and cellular origin. In idiopathic pulmonary fibrosis, CTGF synergizes with TGF-β to amplify proinflammatory signals. It activates the NOX4/ET-1 pathway, exacerbating oxidative stress and ECM deposition, which drives progressive lung tissue destruction (156). In asthma, CTGF levels are positively correlated with MMP-9, TIMP-1, and airway smooth muscle thickness (157). However, during the acute phase of myocardial infarction, CTGF exerts protective effects by reducing inflammation and apoptosis, whereas its overexpression in the chronic phase promotes fibrosis (158, 159). In muscular dystrophy, muscle fiber-derived CTGF does not alter total collagen content but modulates collagen organization, thereby influencing tissue regeneration (160). The functions of CTGF vary significantly across different tissues and diseases, depending on its cellular source and timing of expression. Further research into its precise regulatory mechanisms will provide new insights for developing precision therapeutic approaches for fibrotic diseases.

HGF plays a protective role in tissue remodeling, contrasting with the profibrotic effects of TGF-β. By antagonizing fibrosis, maintaining endothelial barrier integrity, and regulating cell fate, HGF is crucial for multi-organ homeostasis and injury repair (161). Its protective functions are most evident through its dynamic balance with TGF-β. In chronic sinusitis, elevated TGF-β1 drives proliferative remodeling, whereas increased HGF levels are associated with reduced collagen deposition. The ratio between these two factors correlates positively with the severity of remodeling (162). In cardiac injury, HGF reduces scar formation and preserves cardiac function by regulating programmed cell death (163). In the pulmonary vasculature, HGF enhances endothelial barrier stability through activation of Rac signaling and inhibition of the Rho pathway, thus preventing pathological stretch and VEGF-induced barrier disruption (164). These mechanisms underscore the molecular basis for HGF’s role in tissue homeostasis. However, HGF’s effects are not always protective. In certain pathological contexts, it may contribute to pathological remodeling. For instance, in VEGFR inhibitor-resistant lung cancer, stromal-derived HGF induces tumor vascular remodeling through c-MET signaling activation (165). In cervical cancer, despite HGF/c-MET overexpression, HGF levels show no direct correlation with tumor invasion or stromal remodeling, further demonstrating the context-dependent nature of HGF’s function (166). In summary, HGF primarily acts as a protective factor, but its effects are highly dependent on the specific pathological context.

The IGF family, comprising IGF-1 and IGF-2, shares structural similarities with insulin. Together with their binding proteins and receptors, they form a complex regulatory network that governs cell proliferation, differentiation, survival, and metabolism (167). Current research indicates that the role of IGF in tissue remodeling extends beyond merely promoting growth, displaying diversity, context dependence, and intricate regulatory hierarchies. In bone and cartilage, IGF maintains extracellular matrix metabolic homeostasis, and low IGF-1 levels in patients with idiopathic arthritis are associated with abnormal cartilage metabolism (168). In diabetes-related impairments of bone remodeling, exogenous IGF-1 improves bone remodeling by suppressing inflammation and upregulating BMP-2 (169). In the cardiovascular system, IGF-1 exhibits protective effects. Following myocardial ischemia, IGF-1 improves cardiac function through receptor activation (170). Preconditioning with T3 reduces infarct size, inhibits apoptosis and fibrosis, and promotes angiogenesis via the IGF-1/PI3K/AKT pathway (171). In the cerebral vasculature, IGF-1 contributes to adaptive vascular remodeling by maintaining smooth muscle cell and ECM homeostasis (172). However, in the tumor microenvironment, IGF signaling can promote pathological remodeling. In the tumor microenvironment, IGF-1 derived from omental preadipocytes promotes ovarian cancer progression by activating NF-κB signaling (173). Given these diverse roles, future research should focus on disease context-specific and tissue-specific regulation of IGF signaling to achieve precision interventions.

The EGF family signaling network serves as a central regulator of multi-organ remodeling, linking inflammation, metabolic cues, and environmental stimuli to structural changes (174). In airway diseases, sustained EGFR activation promotes smooth muscle thickening and goblet cell metaplasia (175), and drives EMT and mucus secretion via ERα, p38 MAPK, and other pathways (176, 177). Targeting EGFR and MAPK can reduce collagen deposition (126). In the kidney, uremic toxins directly bind to the extracellular domain of EGFR, activating downstream signaling and upregulating MMP-2/9, thereby promoting ECM degradation (178). In the heart, HB-EGF promotes cardiomyocyte hypertrophy and fibroblast proliferation, and its overexpression exacerbates fibrosis and inflammation, contributing to pathological cardiac remodeling (179). In summary, the EGF and EGFR signaling network mediates the conversion of inflammatory signals into structural changes across various organs, responding to metabolic or environmental stimuli to modulate tissue architecture. The ultimate biological effects of this network are highly dependent on specific tissue types and pathological microenvironments. Therefore, future intervention strategies targeting EGF signaling must prioritize tissue specificity and disease stage to achieve precise therapeutic regulation.

## 4 Emerging regulatory mechanisms in tissue remodeling

### 4.1 Non-Smad signaling pathways of TGF-β

Non-Smad signaling pathways of TGF-β play both synergistic and independent roles in fibrotic remodeling, interacting with the Smad pathway to drive abnormal ECM accumulation (180). Key non-Smad signaling pathways downstream of TGF-β include members of the MAPK family, including ERK, JNK, and p38. Their effects are context-dependent. For example, in human dermal fibroblasts, inhibiting ERK or JNK enhances TGF-β1 activation, demonstrating crosstalk between these pathways (181). Similarly, the Prim-O-glucosylcimifugin reduces intestinal fibrosis by inhibiting both TGF-β/Smad and MAPK/ERK signaling, modulating the MMP-1/TIMP-1 balance (115). The Rho/ROCK pathway is another important effector through which TGF-β regulates cytoskeletal dynamics and cell migration. It can be activated directly by TGF-β or triggered by upstream factors like mTORC1 dysregulation, leading to disruption of cell junction proteins and compromised barrier function (182). Non-Smad signaling pathways of TGF-β in cancer has also gained attention. Hamidi et al. found that TGF-β activates the PI3K-AKT pathway to promote cell migration in prostate cancer, independent of TβRI kinase activity and the canonical Smad pathway (183). In inflammatory cardiomyopathy, TGF-β activates the non-canonical kinase TAK1, which then activates the Wnt/β-catenin pathway to drive fibroblast-to-myofibroblast conversion (184). This same signaling axis is involved in myopic scleral remodeling, where Wnt/β-catenin activation downregulates TGF-β1 expression, suppressing collagen synthesis (185). The Wnt pathway’s role in relation to TGF-β is bidirectional, functioning both upstream and downstream, with its specific role determined by the tissue context. Non-Smad signaling pathways of TGF-β also interacts with other pathological pathways. For instance, simultaneous inhibition of the JAK2/STAT3 pathway and canonical TGF-β signaling promotes ECM degradation in liver fibrosis while suppressing autophagy and cell activation (186). In pulmonary fibrosis, Notch signaling activates TGF-β, driving Smad3-dependent EMT through upregulation of TGF-β ligands (187). Zhang et al. demonstrated that knocking down glutaminase expression inhibits both Smad and non-Smad, suppressing proliferation, migration, invasion, and EMT in esophageal squamous cell carcinoma while promoting apoptosis (188). These findings suggest novel therapeutic strategies targeting TGF-β signaling, ranging from anti-fibrosis to anti-tumor metastasis. Despite increasing recognition of the interactions between Smad and non-Smad in TGF-β-induced fibrosis, their precise *in vivo* relationships still require further exploration.

### 4.2 Mechanosensing and mechanotransduction

#### 4.2.1 Integrins, the FAK signaling hub, and the YAP/TAZ signaling pathway

**Figure 3:**
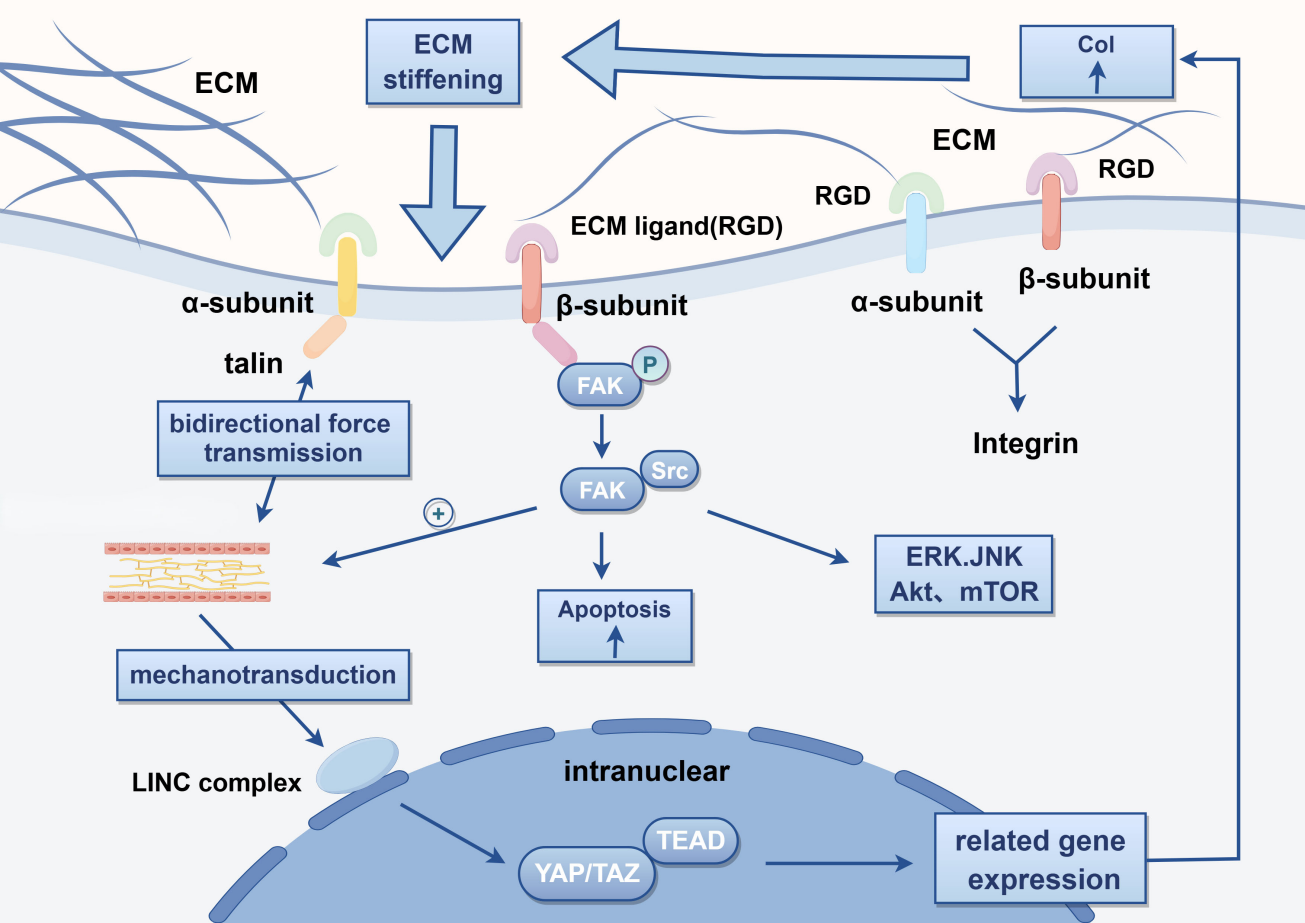
Mechanical signals generated by ECM stiffening are sensed by integrins, relayed through FAK, and transduced via actin stress fibers to YAP/TAZ, driving nuclear transcription. This leads to related gene expression, such as increased Col expression. Increased Col expression leads to ECM deposition and ECM stiffening, thereby exacerbating this process.

Accumulating evidence indicates that mechanical signals derived from the ECM are not merely passive consequences of disease progression but active controllers of tissue remodeling (189). See **Figure 3**. In fibrotic tissues, tumors, and chronically injured organs, progressive ECM stiffening generates sustained mechanical cues that influence cell fate, activation states, and transcriptional programs, thereby profoundly altering cellular behavior (190). Integrins serve as primary mechanosensors linking the ECM to the intracellular cytoskeleton and converting external physical forces into biochemical signals. They recognize specific ECM ligands and form focal adhesion complexes on the cell membrane, thereby establishing continuous mechanical connections between the ECM and the cytoskeleton, enabling bidirectional force transmission and activation of downstream signaling cascades (9). In myocardial infarction, the expression level of αvβ3 integrin predicts remodeling outcomes. Early high expression predicts favorable left ventricular remodeling without adverse structural changes, whereas low expression is strongly associated with progressive ventricular dilation. These findings support a protective role of αvβ3 integrin-mediated mechanotransduction in tissue repair and the suppression of pathological remodeling (191). In contrast, in asthma-associated airway remodeling, mechanical signaling activates latent TGF-β through integrin αvβ5. This process represents a rate-limiting step in regulating TGF-β bioavailability and directly promotes matrix synthesis. Pharmacological inhibition or genetic deletion of αvβ5 attenuates airway smooth muscle thickening, revealing a mechanism by which bronchoconstriction promotes remodeling through αvβ5-mediated activation of TGF-β (192).

Following integrin engagement, FAK undergoes autophosphorylation and recruits Src family kinases to form the FAK-Src signaling complex, which functions as a central mechanotransduction hub. Activation of this axis transmits mechanical signals to multiple downstream pathways, including ERK, JNK, Akt, and mTOR signaling (193). In fibrotic remodeling, FAK can be activated by TGF-β1 in a time- and dose-dependent manner, and this activation precedes the expression of fibroblast activation markers. By driving stress fiber formation, enhancing migration, and inhibiting apoptosis, FAK promotes ECM synthesis and deposition (194). However, the role of FAK in remodeling is highly tissue- and context-dependent. In post–myocardial infarction cardiac remodeling, FBLN7 activates the FAK/AKT pathway through EGFR, promoting fibroblast-to-myofibroblast transdifferentiation and thereby driving fibrosis (195). In contrast, during bone healing, FAK deficiency does not markedly affect osteoblast differentiation, migration, or angiogenesis. Defects in reparative bone formation primarily result from impaired osteoclast attachment and abnormal collagen matrix organization. Moreover, Pyk2 can compensatorily localize to focal adhesions in FAK-deficient osteoblasts, suggesting the existence of signaling compensation mechanisms (196). In summary, although FAK serves as a central mediator of mechanotransduction, its functions differ markedly among tissues and may involve compensatory signaling pathways. Therefore, therapeutic strategies targeting FAK should take its tissue specificity into consideration.

YAP and TAZ function as key transcriptional effectors that convert mechanical signals into gene expression programs. Unlike classical ligand-dependent signaling pathways, YAP and TAZ activity is highly sensitive to cell shape, cytoskeletal tension, and substrate stiffness (197). The activation of YAP/TAZ exhibits time-dependent bidirectional effects in tissue remodeling. During acute liver injury, transient suppression of the Hippo pathway facilitates nuclear translocation of YAP and TAZ, activating target genes that promote hepatic stellate cell activation, transdifferentiation, and synthesis of regenerative ECM. Under chronic injury conditions, however, sustained YAP/TAZ transcriptional activity accelerates fibrotic progression. Persistent activation promotes excessive ECM deposition, ultimately leading to scar formation and progressive tissue stiffening (198). In vascular remodeling associated with pulmonary arterial hypertension, YAP and TAZ act as key drivers of mechanobiological feedback loops in pulmonary artery smooth muscle cells. Their activation is jointly regulated by early ECM stiffening and inflammatory cytokine–mediated non-canonical IκB kinase signaling. This integrated regulation promotes pathological remodeling of pulmonary artery smooth muscle cells (199). These examples demonstrate that the effects of YAP/TAZ depend on the duration of activation and the associated cooperative signaling pathways. Therefore, therapeutic targeting of YAP/TAZ should distinguish between acute and chronic contexts to avoid disrupting their physiological reparative functions.

#### 4.2.2 Synergistic networks between mechanical signals and TGF-β

Mechanical signaling is not only closely associated with the TGF-β pathway but also serves as a key upstream driving factor, indicating a functional coupling between the two. Specifically, mechanical signals directly mediate the activation of latent TGF-β through integrin αvβ5, thereby increasing the local bioavailability of TGF-β (192). Meanwhile, YAP and TAZ act as key mechanical regulators of TGF-β-Smad signaling, participating in signal integration and regulation through multiple mechanisms. First, YAP and TAZ interact with Smad2/3 to regulate their nucleocytoplasmic shuttling. Nuclear exclusion of YAP retains Smad2/3 in the cytoplasm, thereby suppressing TGF-β signaling (200). Second, binding of YAP to activated Smad1 enhances BMP-induced transcriptional activity (201). In addition, reduced YAP/TAZ levels activate AP-1 activity, which subsequently induces Smad7 expression and thereby suppresses TGF-β signaling (202). Together, these mechanisms constitute a regulatory network in which mechanical signaling acts as the initiating factor and TGF-β signaling serves as the central effector. These findings indicate that the regulatory logic of tissue remodeling is not simply a linear activation process. Instead, cells interpret the physical properties of the microenvironment, including matrix stiffness and mechanical stress, and selectively modulate different Smad proteins through YAP/TAZ signaling. This process determines whether TGF-β signaling shifts toward pro-fibrotic or pro-differentiation outputs, ultimately shaping specific cellular behaviors and tissue architecture. Therefore, targeting mechanotransduction pathways may provide an opportunity to intervene in pathological remodeling at its origin, although their complexity requires careful consideration.

### 4.3 Metabolism and remodeling

When dissecting the relationship between metabolic reprogramming and tissue remodeling, a central question arises: are the observed metabolic changes drivers of remodeling, or adaptive responses resulting from the remodeled microenvironment. In the former scenario, metabolic alterations precede and are sufficient to induce remodeling, and blocking these changes can halt the remodeling process. In the latter, metabolic changes occur secondary to structural or functional alterations, and interventions produce only partial effects. The causal roles of different metabolic pathways vary significantly across contexts and therefore require stratified, case-by-case discussion.

#### 4.3.1 Glycolysis

**Figure 4:**
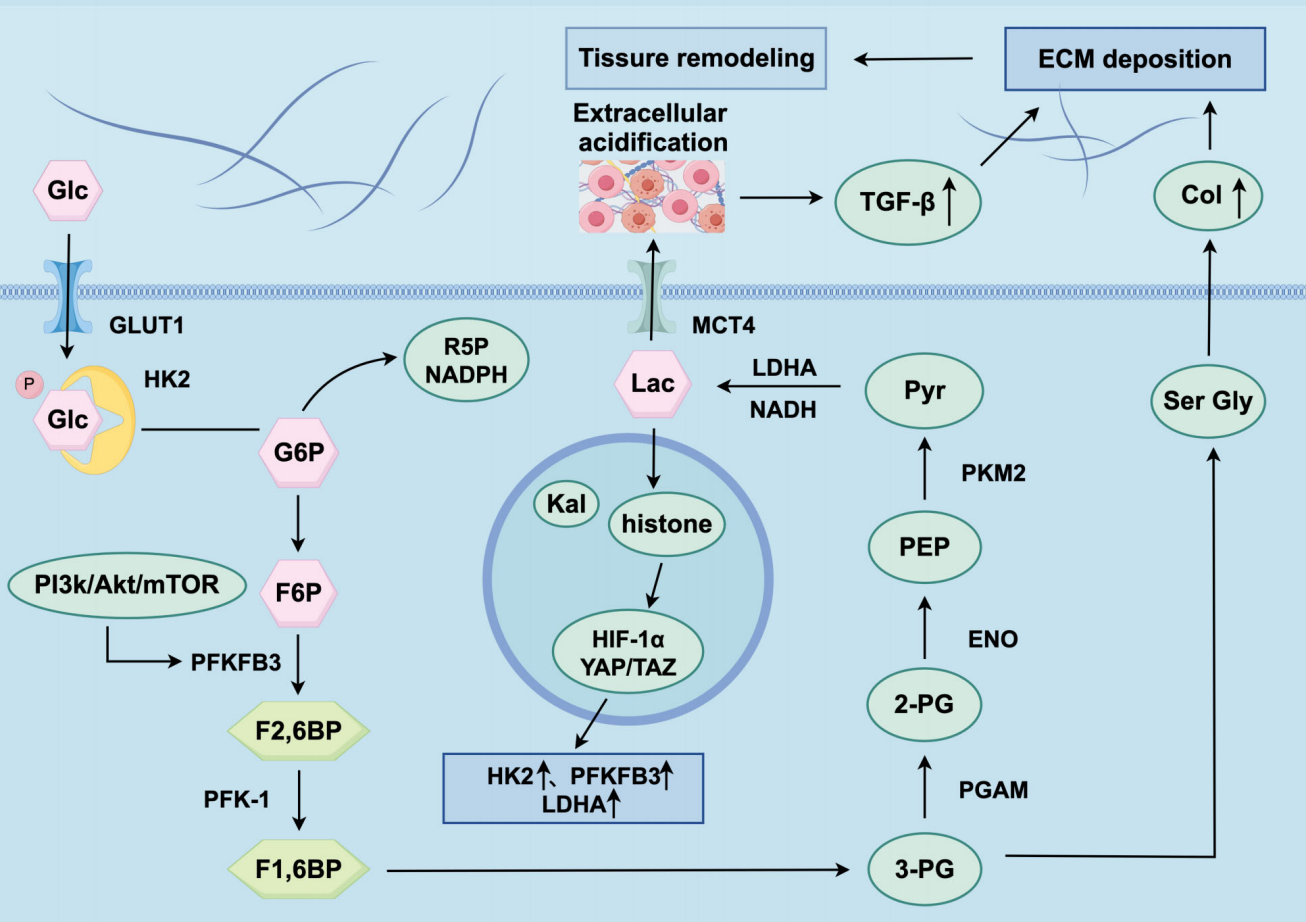
This figure summarizes the integrative model proposed in this review, highlighting the role of the multilevel signaling nexus involving lactate, histone lactylation, HIF/YAP/TAZ, and TGF-β in metabolism-driven fibrosis. On one hand, glycolysis provides glycine for collagen synthesis through the serine synthesis pathway. On the other hand, it generates large amounts of lactate. After being exported from the cell, lactate has dual effects. It acidifies the microenvironment, directly activating TGF-β and promoting fibrosis, and it also enters the nucleus to induce histone lactylation, driving the transcription of specific genes. These genes include not only glycolytic enzymes but also regulators that upregulate or activate transcription factors such as HIF and YAP/TAZ. These factors form a tightly interconnected network with the TGF-β signaling pathway, synergistically amplifying pro-fibrotic signals. These processes ultimately lead to ECM deposition and the progression of tissue remodeling.

The activation of fibroblasts during pathological tissue remodeling is not merely a phenotypic transformation but also involves profound metabolic reprogramming. These metabolic changes reflect cellular activation levels and functionally support sustained ECM synthesis and deposition, linking metabolism with tissue remodeling (10). See **Figure 4**. Activated fibroblasts often shift towards glycolytic pathways for energy production, even in oxygen-sufficient conditions. This metabolic shift rapidly meets energy demands and provides essential intermediates for ECM synthesis (203). Enhanced glycolysis and lactate accumulation form a positive feedback loop, representing a core logic of cross-organ metabolic reprogramming. It is important to note that the causal role of glycolysis in remodeling varies depending on the specific context. As a driving factor, during the initiation stage of fibrosis, TGF-β1 directly upregulates HK2 and PFKFB3 while suppressing their autophagic degradation, and simultaneously regulates Parkin-mediated mitophagy, collectively enhancing glycolysis (204). At this stage, glycolytic activation occurs prior to substantial ECM deposition, and inhibition of the LRRN3/PFKFB3 signaling axis with albendazole can block TGF-β1–induced fibroblast-to-myofibroblast transdifferentiation (205). Enhanced GLUT1-dependent glycolysis is also observed in aged lung fibroblasts and scar-like fibrotic tissues, reinforcing the idea that metabolic alterations promote ECM synthesis during fibrotic progression (206, 207). These findings support glycolysis acting as an upstream driver in early fibrosis. In contrast, within established fibrotic lesions, glycolysis is secondarily enhanced in response to hypoxia, mechanical stress, and inflammatory metabolites. In this context, increased glycolysis represents an adaptive consequence of the remodeled microenvironment, primarily serving to maintain the activated cellular phenotype rather than initiating remodeling. Even so, lactate produced through glycolysis can still amplify fibrosis via a positive feedback loop. Specifically, lactate promotes H3K18la histone modification, thereby upregulating Notch1 expression and driving fibroblast-to-myofibroblast transdifferentiation (208). Macrophages activate H4K12 lactylation via lactate uptake, forming a feedback loop that upregulates TGF-β transcription and enhances fibroblast collagen synthesis (209). Similarly, upregulation of the glycolytic enzyme PKM2 in renal tubular cells leads to lactate accumulation, which promotes TGF-β1 transcription via histone H3K18 lactylation. This process activates macrophage Smad3 signaling, driving macrophage-to-myofibroblast transformation and accelerating renal fibrosis progression (210). These lactate-mediated effects exist in both driving and consequence contexts, but the therapeutic window differs. Early inhibition of glycolysis can prevent the initiation of remodeling, whereas late intervention only partially delays disease progression. Collectively, enhanced glycolysis and lactate accumulation translate metabolic signals into epigenetic alterations through lactylation modifications, highlighting the intrinsic link between metabolism and epigenetic regulation.

#### 4.3.2 Mitochondrial reprogramming

Mitochondrial functional reprogramming is a central metabolic feature of fibroblast activation and fibrotic remodeling. This reprogramming involves more than just a loss of function; it includes adaptive changes such as altered oxidative phosphorylation efficiency, abnormal mitochondrial dynamics, and enhanced ROS signaling (211). The causal role of mitochondrial dysfunction is heterogeneous across different contexts. As a driving factor, in early-stage metabolic heart disease, elevated mitochondrial ROS levels decrease oxidative phosphorylation efficiency and deplete high-energy phosphate compounds. This metabolic disruption precedes myocardial hypertrophy and fibrosis, directly impairing cardiac function (212). Similarly, in renal tubulointerstitial fibrosis, dysfunction in the mitochondrial electron transport chain is a key upstream event in TGF-β1-induced EMT (213). In pulmonary arterial hypertension-associated right ventricular fibrosis, overexpression of PDK1/3 inhibits pyruvate dehydrogenase, reduces oxidative phosphorylation efficiency, and increases mitochondrial ROS. This process sustains a pro-fibrotic phenotype via the DNMT1-HIF-1α epigenetic pathway (214). As a consequence, in chronic fibrosis models, prolonged TGF-β1 stimulation secondarily impairs mitochondrial function. In this context, mitochondrial abnormalities represent downstream outcomes of tissue remodeling and metabolic microenvironment alterations. While inhibition of mitochondrial ROS can partially alleviate fibrosis, it cannot reverse already established ECM deposition. In pulmonary fibrosis, myofibroblast differentiation is accompanied by enhanced glycolysis and altered mitochondrial respiration. This metabolic shift, marked by PFKFB3-mediated glycolytic flux upregulation, succinate accumulation, and reduced oxidative phosphorylation, stabilizes HIF-1α and promotes collagen deposition (215). Overall, altered mitochondrial oxidative phosphorylation and enhanced ROS signaling are common metabolic mechanisms underlying fibrotic remodeling across multiple organs. These changes not only maintain fibroblast activation but also promote ECM deposition via epigenetic modifications and MAPK signaling. Targeting mitochondrial metabolic reprogramming presents a promising strategy for intervening in fibrotic diseases. However, when targeting mitochondrial metabolic reprogramming, it is essential to distinguish between interventions aimed at the initiation phase and those intended for maintenance therapy, as indiscriminate inhibition regardless of context may lead to significant toxicity.

#### 4.3.3 Hypoxia and HIF signaling

Hypoxia is a key microenvironmental factor driving tissue remodeling, primarily through transcriptional reprogramming involving HIF-1α and HIF-2α (216). HIF-1α is a central regulatory node that connects metabolic adaptation to structural remodeling, playing crucial roles in pathological remodeling across various organs by regulating cellular metabolism, angiogenesis, and matrix metabolism (217). HIF-1α typically acts as a driving factor in tissue remodeling, but it can also function as a consequence and maintenance mediator. In pulmonary vascular remodeling, HIF-1α forms a cross-regulatory loop with CD146, which together promote cellular conversion toward synthetic phenotypes, driving vascular remodeling (218). During angiogenesis, HIF-1α stabilizes under acute hypoxic conditions, triggering a metabolic shift toward glycolysis and initiating angiogenesis through endothelial cell activation and ECM remodeling (219). In adipose-derived stem cells, overexpression of HIF-1α couples osteogenesis with angiogenesis via the VEGF-AKT-mTOR signaling pathway. This enhances osteogenic differentiation and promotes bone remodeling and osseointegration around titanium implants, highlighting the regenerative potential of HIF-1α activation in specific repair contexts (220). In established fibrotic tissue, hypoxia resulting from increased cell density and prolonged oxygen diffusion distance is a secondary consequence of structural remodeling, and HIF-1α activation at this stage primarily serves a maintenance rather than initiating role. However, this does not diminish its therapeutic relevance. Downregulation of HIF-1α effectively suppresses the progression of experimental myopia without affecting normal ocular growth, suggesting that it is a key regulatory factor in scleral remodeling in myopia (221). Therefore, HIF-1α exerts a dual role in both driving maladaptive remodeling under pathological conditions and promoting regenerative processes in reparative contexts. Therapeutic strategies targeting HIF-1α should take into account tissue specificity and disease stage. Early inhibition may prevent the initiation of remodeling, whereas late intervention can still suppress feedback-driven maintenance processes.

#### 4.3.4 mTOR signaling pathway

The PI3K-Akt-mTOR signaling pathway is a critical hub for integrating nutrient and growth factor signals. Its sustained activation plays a central role in maintaining fibroblast activation phenotypes and driving pathological remodeling across multiple organs (222). This pathway significantly promotes collagen synthesis and deposition in lung fibroblasts by activating the downstream target PFKFB3, which facilitates aerobic glycolysis (223). Aberrant activation of the mTOR pathway contributes to excessive cell proliferation, resistance to apoptosis, and metabolic reprogramming in pulmonary vascular wall cells, collectively driving structural remodeling of pulmonary arterioles (224). The mTOR inhibitor PP242 induces vascular smooth muscle cell apoptosis and reverses pulmonary vascular remodeling by inhibiting both mTORC1 and mTORC2 in pulmonary arterioles. It also attenuates cardiomyocyte hypertrophy, fibrosis, and right ventricular remodeling by selectively suppressing overactive mTORC1 signaling in right ventricular cardiomyocytes, providing dual tissue protection in both the pulmonary vasculature and the heart (225). Under pathological conditions, activation of the AKT-mTOR pathway directly mediates cardiomyocyte hypertrophy and apoptosis, leading to cardiac dysfunction and tissue fibrosis (226). In airway remodeling, exposure to microplastics disrupts airway epithelial barrier function and induces abnormal HSP90α secretion. This activates the PI3K-Akt-mTOR signaling pathway, which promotes airway smooth muscle cell proliferation, exacerbates inflammatory infiltration, increases mucus secretion, and induces airway hyperresponsiveness, ultimately leading to airway structural remodeling and dysfunction (227). In contrast, AMPK acts as a cellular energy sensor activated under hypertrophic stimulation when energy charge declines. AMPK inhibits mTOR through phosphorylation modifications, attenuating cardiac hypertrophy. This process also involves metabolic reprogramming that suppresses glycolysis and mitochondrial oxidation, improving energy metabolism disturbances (228). In-depth analysis of the interactive regulatory mechanisms within the mTOR signaling network and organ crosstalk will provide important theoretical foundations and molecular targets for developing shared therapeutic strategies aimed at fibroproliferative diseases and pathological remodeling.

### 4.4 ncRNA regulatory network

The importance of ncRNA in fibrosis and tissue remodeling has garnered increasing attention in recent years. As key regulators of gene expression, ncRNA interact with various cellular signaling pathways to precisely modulate cell proliferation, differentiation, migration, and fibrotic responses. The main types of ncRNA include miRNA, lncRNA, and circRNA (11). Through diverse mechanisms, these molecules regulate critical pathways and play pivotal roles in the initiation and progression of various diseases, particularly in the context of pathological tissue remodeling.

#### 4.4.1 miRNA

miRNAs regulate tissue remodeling through post-transcriptional suppression of target gene expression. Their biogenesis involves a series of steps including nuclear processing, nucleocytoplasmic transport, and cytoplasmic maturation, ultimately culminating in functional execution by the RISC complex (229, 230). Numerous miRNAs participate in fibrosis and remodeling, among which the miR-21, miR-29, and miR-200 families are the most representative. miR-21 is markedly upregulated in pathological remodeling across multiple organs and forms positive feedback loops by targeting multiple negative regulatory factors. Following TGF-β1–induced miR-21 expression, upregulated miR-21 promotes ECM synthesis and drives airway remodeling by suppressing Smad7 (231). In cardiac fibrosis, miR-21 activates STAT3 through downregulation of CADM1, thereby promoting fibroblast proliferation (232). In pulmonary vascular remodeling, miR-21-5p suppresses BMPR2 and regulates MMP-7/19 and TIMP-3, leading to ECM imbalance (233). Notably, miR-21 knockout attenuates structural alterations in lung injury models (234). Moreover, innovative delivery strategies can reprogram miR-21 from a pro-fibrotic factor into a reparative regulatory tool, promoting the transition of inflammatory monocytes toward a reparative phenotype (235). These findings suggest that miR-21 serves not only as a central pro-fibrotic regulator but also as a potential therapeutic resource when modulated through precise delivery strategies. miR-29 is a classic anti-fibrotic miRNA that exerts protective anti-remodeling effects across multiple organs by directly targeting collagen synthesis-related genes and inhibiting collagen production (236). In various fibrosis models, downregulation of miR-29 serves as an upstream event that drives profibrotic effects. In bladder outlet obstruction models, c-Myc/NF-κB/SMAD3 suppresses miR-29, resulting in derepression of matrix proteins and subsequent ECM deposition (237). In cyclosporine A–induced cardiac remodeling, TGF-β–Smad3 signaling suppresses miR-29, releasing inhibitory constraints on MMPs and thereby promoting fibrosis (238). In NASH-induced liver injury, reduced miR-29 expression enhances collagen production, and its serum level may serve as an early biomarker of fibrosis (239). In the glaucomatous optic nerve head, dysregulation of miR-29 removes suppression of TGF-β–driven ECM deposition (240). However, in cardiomyocytes, miR-29 directly targets four inhibitors of the Wnt signaling pathway, paradoxically promoting pathological cardiac hypertrophy (241). This paradox highlights that the same miRNA can exert opposite effects in different cell types by targeting distinct regulatory networks, indicating that cell-specific delivery is essential for achieving precise therapeutic intervention. The miR-200 family plays a critical role in maintaining epithelial phenotypes and suppressing EMT, providing protective effects in fibrosis and tumor remodeling across multiple organs. miR-200 suppresses EMT by targeting ZEB1/ZEB2 and maintaining E-cadherin expression (242). In pulmonary fibrosis, downregulation of miR-200 in alveolar type II epithelial cells leads to derepression of ZEB1/2, thereby promoting EMT and cellular senescence, impairing transdifferentiation capacity, whereas restoration of miR-200 reverses fibrotic activity (243, 244). In the tumor microenvironment, miR-200 targets NRP2 to block secretion of factors such as VEGF-D, thereby inhibiting the pro-migratory and M2-polarizing effects of cancer-associated fibroblasts (245). Moreover, miR-200 expression is subject to HDAC-mediated epigenetic silencing. HDAC induces transcriptional repression by deacetylating the miR-200 promoter region, promoting tumor progression (246). In liver fibrosis models, upregulation of miR-200 reduces ECM deposition and oxidative stress by inhibiting the TGF-β1-Smad3 signaling pathway, thereby attenuating fibrosis and preserving liver tissue integrity. This highlights miR-200’s function as a negative regulator of the TGF-β1-Smad3 axis in suppressing liver fibrosis progression (247). Restoration of miR-200 expression or reversal of its epigenetic silencing represents a shared anti-fibrotic strategy across multiple organs. In summary, miRNAs constitute an important dimension of epigenetic reprogramming. Their functions are highly dependent on cell type and target gene networks, allowing the same molecule to exert bidirectional effects, including pro-fibrotic or anti-fibrotic, as well as protective or deleterious actions. Precise delivery and cell-specific regulation therefore represent the central challenges for future miRNA-based therapies.

#### 4.4.2 lncRNA

lncRNAs participate in pathological remodeling across multiple organs by regulating ECM metabolism, fibroblast activation, and cell fate through ceRNA mechanisms (248). Their functions exhibit bidirectional effects, including both promotive and inhibitory roles, and are highly dependent on the specific lncRNA involved and the disease context. The lncRNA MALAT1 mediates pathological remodeling in various vascular diseases by sponging specific miRNA. By competitively binding miR-145-5p, MALAT1 relieves inhibition of HK2, promoting vascular smooth muscle cell proliferation, migration, and phenotypic transformation, which drives hypertension-related vascular structural changes (249). Additionally, MALAT1 sponges miR-124-3p, relieving its inhibition of KLF5, thereby stimulating pulmonary artery smooth muscle cell proliferation and cell cycle progression, contributing to pulmonary vascular remodeling (250). Downregulation of MALAT1 relieves suppression of the miR-125a-5p/STAT3 axis, promoting progenitor cell differentiation into smooth muscle cells and thereby leading to vascular stenosis (251). PVT1 functions as a ceRNA by sponging miR-30a, upregulating Beclin-1 expression and activating autophagy. This promotes cardiomyocyte fibrosis-related protein expression, exacerbating ventricular remodeling and myocardial fibrosis (252). In the myocardial infarction border zone, PVT1 sponges miR-181a, preventing degradation of target genes including TNF, Met, Itgam, and Bst1, and forms a co-expression regulatory axis with Bst1, collectively contributing to pathological remodeling in the infarct border zone (253). In contrast, GAS5 exerts protective effects by inhibiting pathological remodeling in lung tissue. GAS5 is downregulated in COPD, where it suppresses inflammation and fibroblast activation and attenuates airway remodeling by sponging miR-217-5p and upregulating PTEN (254). In chronic thromboembolic pulmonary hypertension, GAS5 acts as an upstream regulator of miR-382-3p, inhibiting smooth muscle proliferation, reducing pulmonary arterial pressure, and alleviating vascular thickening (255). Collectively, lncRNA play dual roles in tissue remodeling through ceRNA mechanisms, either promoting or inhibiting these processes. Therapeutic strategies targeting lncRNA must precisely define their functional direction in specific disease contexts and develop tailored interventions.

#### 4.4.3 circRNA

circRNA, an emerging class of ncRNA, play pivotal regulatory roles in tissue remodeling through diverse molecular mechanisms. circASH2, which is absent in hepatocellular carcinoma, inhibits tumor metastasis by enhancing the liquid-liquid phase separation of nuclear YBX1 protein. This process accelerates splicing and degradation of TPM4 transcripts, reducing levels of the actin-binding protein TPM4 and altering tumor cell cytoskeletal structure (256). circPTK2 undergoes m6A modification mediated by the methyltransferase METTL3 and is exported from the nucleus via EIF4A3. In the cytoplasm, it functions as a sponge for miR-484 and miR-125a-3p, upregulating YAP1 and FYN expression while activating STAT3 signaling. This cascade drives fibroblast activation and promotes pulmonary fibrosis progression (257). circGUCY1A2, downregulated in hypoxia-induced pulmonary arterial hypertension, binds directly to Ser1132 and Ser1145 residues of COL3A1, inhibiting O-glycosylation and reducing protein stability. This leads to decreased osteopontin expression and prevents pulmonary artery smooth muscle cells from converting toward synthetic phenotypes, thereby inhibiting pulmonary vascular remodeling (258). These examples illustrate that circRNA exert regulatory functions far beyond conventional miRNA sponging. Their networks encompass post-transcriptional processing, epigenetic modification, and protein stability regulation across multiple levels. Systematic investigation of circRNA mechanisms under specific pathological conditions will provide a crucial theoretical foundation for developing precision therapeutic strategies targeting tissue remodeling.

## 5 Translational strategies targeting shared remodeling pathways

### 5.1 Therapeutic opportunities and the risks of pleiotropic inhibition

Pathological tissue remodeling represents a common core pathological process underlying chronic fibrotic diseases, airway disorders, myopia, and cardiovascular lesions. The regulatory targets involved exhibit marked pleiotropy, crosstalk, and tissue specificity. Pleiotropic signaling pathways, including TGF-β/Smad, PI3K/AKT/mTOR, and YAP/TAZ, participate not only in physiological repair and homeostasis maintenance but also in pathological ECM deposition, fibroblast activation, EMT, and structural tissue remodeling. Although direct inhibition of these pathways can suppress fibrotic remodeling, it may also interfere with physiological repair, disrupt tissue homeostasis, and induce off-target effects and compensatory activation. For example, systemic inhibition of TGF-β may impair post-injury tissue repair, cause immune dysregulation, and disrupt vascular development, whereas mTOR inhibition may disturb the balance of cellular metabolism and proliferation. These risks limit the clinical translation of single-target interventions and provide a theoretical rationale for combinational targeting strategies.

### 5.2 Combination therapeutic targets

Simultaneous targeting of multiple shared remodeling pathways may achieve synergistic efficacy, reduce toxicity, and enable precise stratification and dynamic monitoring through therapeutic biomarkers. These strategies can be summarized from two major perspectives. First, concurrent intervention in both biochemical signaling pathways and mechanotransduction pathways involved in remodeling can disrupt the pathological positive feedback loop of signal activation, matrix stiffening, and signal amplification, thereby synergistically terminating fibrosis progression at multiple levels (192, 200). Second, the combined use of multi-kinase inhibitors targeting PDGFR/VEGFR/FGFR together with regulators of matrix metabolism can simultaneously block multiple pro-proliferative and pro-vascular remodeling growth factor receptor axes while restoring the balance between MMPs and TIMPs. This approach enables concurrent suppression of excessive mesenchymal cell activation and restoration of ECM homeostasis (8, 67, 74, 145). These combinational strategies have demonstrated clear synergistic effects across multiple organ remodeling models and exhibit substantial potential for clinical translation (198, 202, 259).

### 5.3 Biomarkers of therapeutic response

Biomarkers are central tools for achieving precision intervention. The TGF-β superfamily represents a core pathway driving pathological tissue remodeling, and its active molecules are among the most representative indicators for therapeutic monitoring. Levels of activated TGF-β1 can directly reflect the efficacy of interventions targeting the TGF-β pathway. In diseases such as asthma, pulmonary fibrosis, and myopia, reduced TGF-β1 levels in body fluids are strongly associated with decreased ECM deposition and attenuation of airway or scleral remodeling (17, 22, 260). The signaling intensity of different BMP isoforms may also indicate the degree of remodeling in specific organs (28). Increased expression of BMP-2, BMP-4, and BMP-7 can serve as inverse indicators of restoration of protective reparative pathways and has clear relevance in therapies targeting bone remodeling, scleral remodeling, and cardioprotection (50, 54, 55). Among matrix metabolism–related biomarkers, MMP-2 and MMP-9 are key enzymes involved in collagen and basement membrane degradation, and suppression of their activity indicates correction of abnormal ECM degradation in tissues such as the sclera and airway (78, 80). Restoration of the MMP-9/TIMP-1 ratio may indicate alleviation of airway remodeling (70, 157). Regarding immune-inflammatory biomarkers, reductions in Th2-associated cytokines, including IL-4, IL-5, and IL-13, suggest effective blockade of upstream inflammatory drivers in allergic diseases such as asthma (86, 88). Decreased IL-17A levels indicate restoration of Th17/Treg balance and may predict improvement of vascular, joint, and airway remodeling (93, 261). Downregulation of M1-associated factors such as TNF-α, IL-1β, and IL-6 suggests suppression of inflammation-mediated fibroblast activation (167, 169). Notably, reduced local ocular IL-6 levels can directly reflect inhibition of the scleral remodeling axis in myopia and may indicate attenuation of axial elongation progression (171).

In addition, suppression of YAP/TAZ nuclear translocation indicates inhibition of tissue remodeling and is applicable for monitoring fibrosis in the liver, pulmonary vasculature, and airway (205, 206). Downregulation of HIF-1α suggests suppression of hypoxia-driven ECM degradation and myofibroblast transdifferentiation and has specific monitoring value in myopic scleral remodeling and pulmonary vascular remodeling (221). Among non-coding RNA biomarkers, miR-21, a classical pro-fibrotic miRNA, can directly reflect inhibition of the TGF-β pathway and reduced ECM synthesis when downregulated, making it suitable for monitoring remodeling across multiple organs, including the lung, heart, vasculature, and airway (231, 232). Restoration of the miR-29 family indicates suppression of collagen synthesis and recovery of ECM homeostasis and may serve as a positive predictive biomarker in liver, cardiac, and bladder fibrosis (236, 237). Re-expression of the miR-200 family signifies inhibition of EMT and maintenance of the epithelial phenotype, representing an important biomarker for pulmonary fibrosis and tumor microenvironment remodeling (242, 243). Among lncRNAs, downregulation of MALAT1 and PVT1, together with upregulation of GAS5, respectively indicate alleviation of pathological remodeling in the vasculature, heart, and lung (249, 252, 254). CircRNAs such as circPTK2 and circGUCY1A2 can precisely reflect fibroblast activation and the therapeutic response of vascular remodeling interventions (257, 258). Collectively, combinational therapeutic strategies targeting pleiotropic shared pathways, together with the clinical application of therapeutic biomarkers, establish an efficient translational framework linking fundamental mechanisms to clinical practice. This integrated approach provides a unified and feasible therapeutic paradigm for multisystem pathological remodeling disorders and substantially enhances the translational value and precision of anti-remodeling therapies.

## 6 Summary and outlook

Tissue remodeling represents a fundamental adaptive mechanism by which organisms maintain homeostasis and respond to injury, whereas its dysregulation drives pathological processes such as fibrosis. The major conceptual advance of this review lies in the first systematic integration of classical signaling pathways with emerging regulatory networks, revealing a shared core molecular logic underlying pathological remodeling that is profoundly shaped by organ specificity and cellular context. This framework moves beyond the traditional linear view of remodeling mechanisms and instead emphasizes a multilayered and dynamically interactive regulatory model, providing a new perspective for understanding the unified yet opposing relationship between tissue repair and fibrosis.

Nevertheless, the field still faces two major unresolved challenges. First, the context-dependent effects of the same signaling pathways across different cell types remain incompletely understood, resulting in variable therapeutic outcomes when these pathways are targeted. Second, the interplay among mechanical signaling, metabolic reprogramming, and epigenetic regulation remains insufficiently defined. In particular, how mechanical cues reshape metabolic enzyme programs through YAP/TAZ signaling, and how metabolic intermediates direct epigenomic modifications, are still poorly understood. These knowledge gaps hinder the development of effective upstream regulatory strategies. Future research focusing on these two critical gaps, together with the development of multimodal and single-cell-resolution analytical approaches, may enable a transition from merely describing pathological remodeling to precisely reprogramming reparative processes. Such advances could provide genuine translational breakthroughs for chronic progressive diseases.
